# Development and Evaluation of the Validity and Reliability of the Leading and Managing Care Pre-Registration Nursing Student Assessment Tool

**DOI:** 10.1177/23779608211000259

**Published:** 2021-03-31

**Authors:** John Unsworth, Andrew Melling, Debra Porteous

**Affiliations:** 1 Department of Nursing, Midwifery and Healthcare, Northumbria University, Newcastle-upon-Tyne, United Kingdom; 2 Faculty of Health & Life Sciences, Northumbria University, Newcastle-upon-Tyne, United Kingdom

**Keywords:** competence, leadership, management, student nurses, assessment tool

## Abstract

**Background:**

Clinical nursing leadership influences patient safety and the quality of care provided. Nurses at all levels require leadership and management skills. Despite recognition of the importance of leadership, student nurses often feel ill prepared to make the transition to Registered Nurse and struggle with prioritisation and delegation. In order to standardise student experience and promote the development of skills and attributes, a leadership and management competency assessment was developed and implemented.

**Aims:**

This study aimed to identify the constructs that should be part of an assessment of student nurse competence in relation to clinical nursing leadership, and to evaluate the tool’s reliability.

**Method:**

The first phase was to construct the competency assessment tool, using a mixture of deductive methods, including literature and expert review. Second, psychometric evaluation of the tool, including tests to examine its internal consistency and reliability, comparing test and retest reliability, exploratory factor analysis and generalisability theory analysis to identify reliability and sources of error.

**Results:**

Five attributes were identified for inclusion in the tool alongside a scale of competence. 150 assessments were conducted with an average time between each assessment of three days. The results show that the tool was consistent over time with no significant difference in the mean scores. The Cronbach alpha was 0.84 and the tool had good internal consistency. The results of the factor analysis revealed loading onto a single construct. Generalisability theory analysis revealed 0.90 global reliability, with students accounting for the majority of the variation in scores.

**Conclusions:**

The Leading and Managing Care assessment tool represents a valid and reliable assessment of student nurse competence to lead care delivery. Use of the tool during practice placement allows for a structured approach to the development of skills around prioritisation, management of resources, communication and the management of risk.

## Introduction

Clinical nursing leadership is widely acclaimed as a major influence on both patient safety and the quality of care delivered (Brown et al., 2015; Cook & Leathard, 2004). The World Health Organization (2020) has recognised the need for leadership development among newly qualified nurses globally. Indeed, strategic leadership is one of four goals of the ICN Strategic Plan 2019—2023 to meet current and future health care needs (International Council of Nurses, 2019). In addition, the National Health Service in England acknowledges the requirement for strong clinical nursing leadership, describing how health services need leaders at all levels (Department of Health and Social Care, 2018). The importance of leadership at all levels in nursing has also been acknowledged in the United States of America (Heller et al, 2004; Smith-Trudeau, 2016) as central to nursing recruitment and retention, as well as influencing the quality and safety of the care delivered. The leading and organisation of care is a significant part of a registered nurse’s role that includes prioritisation, assessing risk, communication and delegation of work to others. As a result, newly qualified nurses require some experience of leading and managing clinical care at the point of registration even though they will be regarded at this stage as a novice clinical leader (Sherman & Bishop, 2007). In their qualitative interviews with 12 newly qualified nurses, Ekstrom and Idvall (2015) found that many described experiencing problems fulfilling their clinical leadership role, felt ill-prepared to co-ordinate other team members, and struggled to manage resources, prioritise care needs and delegate appropriately. Hendry and Walker (2004) identified similar issues in their descriptive literature review, in which they found that newly qualified nurses found prioritisation a difficult skill and this directly affected their ability to solve problems and make decisions. Several factors were cited as contributing to such difficulties, including the experience of the nurse, the patient’s condition, and the availability and management of resources. In addition, it has been suggested that newly graduated nurses are not ready for leadership roles (Al‐Dossary et al., 2016). It is imperative to understand the nature of nurse preparation for leadership roles to enable and empower undergraduate nursing students to be effective future leaders.

The concept of clinical nursing leadership has not been well defined (Cook, 2001). Ha and Pepin (2018) describe how leadership is a competency demonstrated in clinical care by nurses who are working with and influencing others to provide safe and high quality care. For the purpose of this study, we expanded this definition and identified that clinical nursing leadership relates to a set of competencies, which nurses use to co-ordinate a team to deliver safe, timely and effective care to patients. The competencies include team working and communication, prioritisation of care needs, delegation, and the identification and management of risk.

A number of studies have explored the educational preparation of nursing students in terms of clinical nursing leadership. Ha and Pepin (2018) conducted a qualitative evaluation of an education intervention in the first year of an undergraduate programme. The intervention-included analysis of leadership behaviours in others using pre-prepared videos of leaders at work. The evaluation revealed the value students placed on being able to analyse and observe positive role models. The authors argue that leadership education needs to occur over time using a mixture of intentional and non-intentional approaches in the classroom, as well as experience from clinical placements. Francis-Shama (2016) conducted a UK-based grounded theory study to explore student nurses’ perceptions of leadership prior to qualifying. They found that students valued positive leadership role models but poor clinical learning environments had a negative impact on the students’ perceptions of leadership. The study proposed that programmes could be enhanced in terms of leadership preparation and the development of clinical settings. A study in Brazil by Leal et al. (2017) identified that while leadership and management skills could be learnt and developed during teaching and placement experience, students felt ill prepared for their role as a newly qualified nurse, particularly around the deployment and management of resources in the clinical setting. Scammell et al. (2020) identified that key to the development of strategic leadership within nursing students was dependent on expert input from both academic‐ and practice‐based educators.

Leal et al. (2017) suggest that nurse education programmes could enhance both the theory and practical elements of clinical nursing leadership development. This led to an examination of how practice experience might be standardised to ensure all nurses have an opportunity practice skills associated with the management and leadership of clinical care.

[Name redacted] University offers a 20-week internship placement in the final year of its undergraduate nursing degree programme. The placement runs alongside a module designed to prepare students for working life by exploring concepts of leadership and management. The module includes a range of teaching and learning approaches, including lectures, seminars and interactive case study-based approaches, such as a tabletop workshop around managing resources on a fictional ward. In addition, students undertake a ‘real time’ ward-based simulation exercise, where they are required to lead a shift in the simulation ward from the handoff from night staff through to lunchtime. The simulation is designed to explore, in an experiential learning format, the practical application of leadership skills associated with the prioritisation, delegation, and co-ordination and supervision of care (Murray et al., 2016). Further, during the student’s internship placement, many wards, teams and departments encouraged them to take a leadership role during a shift, either taking responsibility for the entire ward or department, or leading a sub-team for a cohort of patients. This experience was regarded as highly valuable as it allowed students under supervision to experience first-hand aspects of clinical nursing leadership. As part of a curriculum review, the programme team decided to make this leadership opportunity a requirement for all internship placements. At the same time, they devised a new assessment tool, Leading and Managing Care (LMC), to assist practice educators to assess the student’s ability to perform the competencies associated with clinical nursing leadership. This article provides a detailed exploration of the development of the LMC assessment tool and its subsequent psychometric testing in a practice setting.

### Study Questions

The study aimed to address the following questions:

1. Which constructs would an assessment of competence related to clinical nursing leadership for undergraduate nursing students include?
2. Is the assessment of clinical nursing leadership using the LMC tool a valid and reliable assessment when used in the final placement to assess undergraduate nursing students?

### Study Design

The study consisted of two phases of instrument development (Tay & Jebb, 2017), namely:

1. Instrument development and construction
2. Psychometric evaluation of the instrument in the clinical setting

#### Phase 1: Instrument Development and Construction

Instrument development involved three stages, based on a deductive approach to identifying constructs (Tay & Jebb, 2017). The first stage was to identify the construct(s) being assessed using the tool. The LMC tool was designed for use with undergraduate nursing students in the clinical setting. In the final part of their programme, the students undertake an extended ‘internship’ placement for 20 weeks. Towards the end of this placement, prior to qualification, students increasingly take responsibility for leading and managing patient care under the supervision of the nurse in charge. To assist with the development of the constructs/attributes being assessed, a scoping literature review was undertaken to identify what attributes and behaviours are considered as elements of clinical nursing leadership within published work. The second stage involved expert review by clinicians and educators drawn from a range of specialities. Finally, stage three involved the mapping of the tool to the Nursing and Midwifery Council’s Standards of Proficiency for Pre-Registration Nursing Education Programmes (Nursing and Midwifery Council [NMC], 2018).

#### Phase 2: Psychometric Evaluation of the Assessment Tool in the Clinical Setting

Tay and Jebb (2017) describe how it is vitally important to be clear about the purpose of the scale before seeking to examine its psychometric properties. The LMC assessment tool is used by practice educators to judge whether the NMC’s Standard of Proficiency for Pre-Registration Nursing related to leadership have been achieved. A preliminary analysis requires a sample of between 100-200 assessments, while a confirmatory analysis requires a minimum of 300 assessments (Tay & Jebb, 2017). To assess the scale’s psychometric properties, a reliability analysis will be performed using the Statistical Package for the Social Sciences (SPSS) version 25. This will review the internal consistency and the correlation between the assessment tool items and identify whether any item should be removed from the assessment tool. Following this, the data from the two assessment periods will be analysed using a t-test, to see if there is any statistical difference between the first and second test occasion. An exploratory factor analysis (Morgado et al., 2018) was performed to review the construct validity of the assessment tool. Finally, a Generalisability Theory (Bloch & Norman, 2012) study was conducted using EduG software (Cardinet et al., 2010) to identify the global reliability and sources of error. Generalisability theory is a useful method of reviewing variance in assessment tools, because rather than simply providing a view about whether or not a tool is reliable, it identifies potential sources of error.

#### Sample

The participants in this study were drawn from a cohort of adult, mental health and children’s nurses from the Master of Nursing/Registered Nurse programme. The Master of Nursing is a two-year programme for individuals with prior healthcare experience and a health-related degree. n = 75 participants were assessed as part of their approved programme. Student could opt out of having their secondary data used in this study by completing an opt out form in the study information sheet. None of the students opted out and all the data was included.

#### Ethical Approval

The University’s Ethical Review Committee approved this project. The data was generated irrespective of the research, as it formed part of the assessment processes of an approved educational programme. Given that the study used secondary data, participants were invited to have their data processed as part of this research.

## Results

### Instrument Development and Construction

The first stage of instrument development (Tay & Jebb, 2017) involved identifying the constructs to be assessed by conducting a review of the literature. A scoping review was performed to identify the attributes and behaviours associated with clinical nursing leadership. The review searched literature between 2008 and 2018 using EBSCOhost, which among other databases searches CINAHL (Cumulative Index of Nursing and Allied Health Literature) and PsychINFO (which indexes literature related to the field of psychology). The search strategy was limited to peer-reviewed publications in academic journals. Search terms included ‘clinical leadership’, ‘clinical competence’ and ‘leadership education’. The inclusion criteria were: papers had to be published in English and specifically refer to attributes, behaviours or competencies associated with clinical leadership. The search revealed 77 published articles. 11 papers met the inclusion criteria and were reviewed to identify attributes and behaviours. Table 1 summarises the papers and the identified attributes and behaviours, revealing 16 individual items identified. These 16 attributes and behaviours were then reviewed by an expert panel in stage two of the instrument development and construction.

**Table 1.** Identifying Attributes and Behaviours From the Literature.

Citation	Attributes and behaviours	Study information
Hansten (2011)	Delegation	Opinion paper
	Supervision	
		
Lekan et al. (2011)	Supervision	Narrative analysis of
	Delegation	reflective journal entries
	Communication	
		
Satu et al. (2013)	Teamwork	Systematic review
Organisation and management of care		
Delegation		
Decision making		
		
Nilsson et al. (2014)	Observation and responding to risk	Psychometric evaluation
	Patient safety	
	Communication	
	Quality of care	
	Leading a team	
		
Brown et al. (2015)	Management and organising care	Scoping literature review
	Communication	
	Delegation	
	Teamwork	
	Safety and Quality	
	Prioritisation	
	Decision making	
		
Demeh and Rosengren (2015)	Role model	Qualitative interviews with
	Safety and quality	nursing students
	Setting the direction	
		
Ekstrom and Idvall (2015)	Co-ordinating work	Qualitative perceptions of
	Quality and safety	newly qualified nurses
		
Brown et al. (2016)	Risk management	Survey to identify the
	Dealing with change	leadership elements of the
	Conflict management	curriculum
	Supervision of others	
		
Gamble (2017)	Decision making	Evaluation of the value
	Communication	of simulation in leadership
	Time management	development
	Prioritisation skills	
		
		
		
Gunawan et al. (2019)	Assignment of staff	Management and
	Decision making	leadership scale
	Role model	development and testing
	Communication	
		
Scammell et al. (2020)	Communication	Review of the literature
	Role model	
	Patient safety	
		
		

Stage two of the development process involved a review of the items and early drafts of the tool by panels of experts. Three panels were held, with 32 experts drawn from academic staff, lead nurses for education and practice educators. Individuals came from a range of different fields of practice, including adult, children’s and mental health nursing. In addition, a range of clinical specialisms, including acute care, community practice and critical care departments were represented. The expert panels were asked to review the attributes and behaviours identified from the literature and to consider which were appropriate to assess among final year nursing students on placement, and to identify potential duplicate items. Of the 16 attributes, five were selected for inclusion in the tool (Definitions for each attribute can be found in Appendix 01) and prior to, these were:

- Communication
- Management and co-ordination of resources
- Risk assessment and control, including safeguarding
- Prioritisation to ensure timely care delivery
- Delegation to other staff

The expert panel felt that the management and co-ordination of resources included team working, and organising and assigning staff as well as an element of supervision. They felt that patient safety and quality management were inherent in all the attributes and were therefore regarded as global concepts. In addition, as the LMC assessment tool was used alongside another tool, which examined patient care delivery (Unsworth et al., 2020); they felt it was unnecessary to assess clinical decision-making.

**Figure 1.**
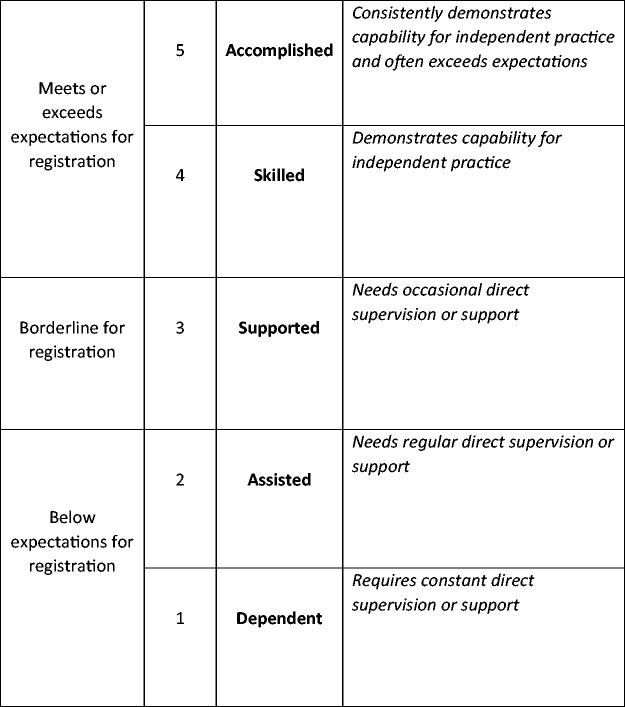
The Scale of Competence Used Within the Assessment Tool (Adapted From Bondy, 1983).

The assessment tool would include the five attributes identified by the expert panel, with each attribute, being assessed using a scale of competence (Figure 1), based on the scale developed by Bondy (1983) but adapted by the expert panel to fit with practice placements in the UK context. Bondy (1983) originally devised a five-item scale using the labels: dependent, marginal, assisted, supervised and independent. The expert panel believed that ‘supervised’ and ‘independent’ were problematic for use in a practice placement context, as all students are supervised irrespective of their stage on the programme and they are not allowed to practice entirely independently until they registered. The scale was adapted by adding a criterion detailing the level of support the student required; this provided a behavioural anchor for the assessor to identify the appropriate level of performance. An additional sixth item, ‘accomplished’ was added at the top of the scale, above ‘exceeds expectations’, providing an incentive to extend beyond the minimum level of competence for registration. Finally, the words ‘independent’ and ‘supervised’ were changed to ‘skilled’ and ‘supported’. Students deemed to be skilled demonstrated the capability for independent practice although they were not truly independent at the time of assessment.

The tool was printed on NCR (No Carbon Required) paper with two copies, allowing the collection of data and for a copy to be retained by the student. As well as recording performance on the scale of competence against each attribute, practice educators could provide qualitative feedback on the form, including areas for future development.

The third stage of assessment tool development and construction was to map the tool against the Standards of Proficiency for Pre-Registration Nursing programmes produced by the Nursing and Midwifery Council (NMC, 2018) in the UK. Table 2 details the 11 proficiencies that are assessed by the tool. These proficiencies are broadly similar to competency statements produced by other regulators in Canada (Canadian Council of Registered Nurse Regulators, 2018), Australia (Nursing and Midwifery Board of Australia, 2006) and Singapore (Singapore Nursing Board, 2018).

**Table 2.** Mapping of the Leading and Managing Care Assessment Tool to Nursing and Midwifery Council Proficiencies (NMC, 2018)

*Platform 1*Being an accountable professional	1.11 communicate effectively using a range of skills and strategies with colleagues and people at all stages of life and with a range of mental, physical, cognitive and behavioural health challenges (NMC, 2018, p. 9).
*Platform 4*Providing and evaluating care	4.9 demonstrate the knowledge and skills required to prioritise what is important to people and their families when providing evidence-based person-centred nursing care at end of life including the care of people who are dying, families, the deceased and the bereaved (NMC, 2018, p. 18).
*Platform 5*Leading and managing nursing care and working in teams	5.3 understand the principles and application of processes for performance management and how these apply to the nursing team (NMC, 2018, p. 20). 5.4 demonstrate an understanding of the roles, responsibilities and scope of practice of all members of the nursing and interdisciplinary team and how to make best use of the contributions of others involved in providing care (NMC, 2018, p. 20). 5.5 safely and effectively lead and manage the nursing care of a group of people, demonstrating appropriate prioritisation, delegation and assignment of care responsibilities to others involved in providing care (NMC, 2018, p. 20). 5.6 exhibit leadership potential by demonstrating an ability to guide, support and motivate individuals and interact confidently with other members of the care team (NMC, 2018, p. 20).
*Platform 6*Improving safety and quality of care	6.2 understand the relationship between safe staffing levels, appropriate skills mix, safety and quality of care, recognising risks to public protection and quality of care, escalating concerns appropriately (NMC, 2018, p. 22). 6.5 demonstrate the ability to accurately undertake risk assessments in a range of care settings, using a range of contemporary assessment and improvement tools (NMC, 2018, p. 22). 6.10 apply an understanding of the differences between risk aversion and risk management and how to avoid compromising quality of care and health outcomes (NMC, 2018, p. 23). 6.11 acknowledge the need to accept and manage uncertainty, and demonstrate an understanding of strategies that develop resilience in self and others (NMC, 2018, p. 23). 6.12 understand the role of registered nurses and other health and care professionals at different levels of experience and seniority when managing and prioritising actions and care in the event of a major incident (NMC, 2018, p. 23).

Completed assessment forms were returned by 75 students and data from a total of 150 assessments (two forms from each student) were analysed. Each student was assessed on two occasions by the same practice educator. The mean duration between the first assessment and the second was three days (Standard deviation 1.414214). The data were entered into the SPSS programme and analysed using a paired sample t-test to identify whether there was a statistically significant difference between the scores on assessment one and assessment two. Table 3 shows the means and standard deviation for each assessment component. The mean scores are very similar and analysis revealed no statistically significant difference in scores between assessment one and assessment two in any component.

**Table 3.** Paired Sample t-Test Results for Each Assessed Component.

Assessed component	Mean	N	Standard deviation	Paired sample t-test result
Communication first assessment	4.69	75	0.51	t(74) = 0.59, *p =* 0.552
Communication second assessment	4.65	75	0.47	
Manage resources first assessment	4.24	75	0.06	t(74) = −1.00, *p =* 0.321
Manage resources second assessment	4.30	75	0.06	
Risk assessment first assessment	4.25	75	0.65	t(74) = −0.86, *p =* 0.388
Risk assessment second assessment	4.32	75	0.52	
Prioritisation first assessment	4.52	75	0.57	t(74) = −0.39, *p =* 0.698
Prioritisation second assessment	4.54	75	0.55	
Delegation first assessment	4.21	75	0.06	t(74) = −1.47, *p =* 0.146
Delegation second assessment	4.30	75	0.06	

**Figure 2.**
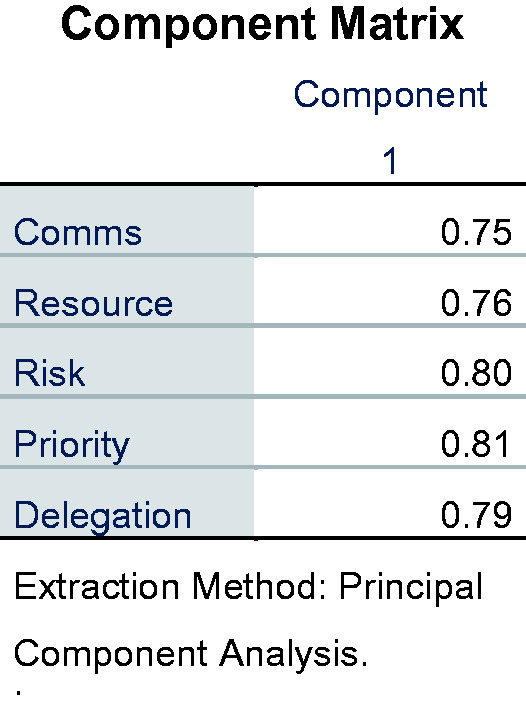
Component Matrix.

**Figure 3.**
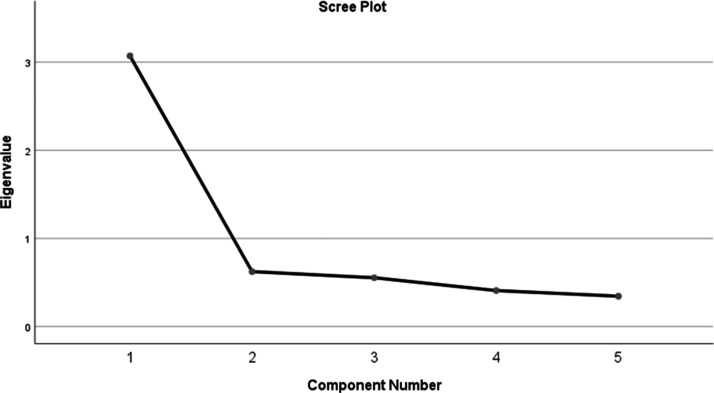
Factor Analysis Scree Plot.

**Figure 4.**
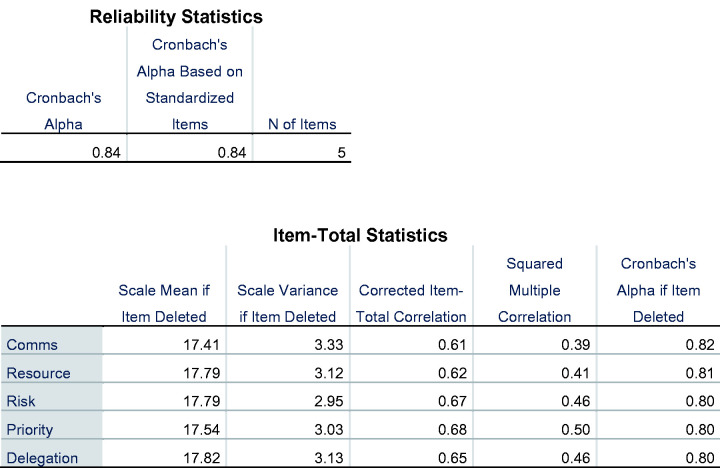
Cronbach Alpha and Correlation.

Exploratory factor analysis was performed to determine the Kaiser-Meyer-Olkin Measure of Sampling Adequacy, which was 0.829. This suggests that the sample was adequate for factor analysis. The Bartlett’s Test of Sphericity (ê−^2^ (10) = 280.944, *p* – 0.000) indicates that this is significant and that the factor analysis is appropriate. The factor analysis uses maximum likelihood with oblique rotation as the method of extraction. This identifies a single factor (component) onto which all variables are loaded (Figure 2). The scree plot (Figure 3) confirms this and the single factor suggests that the tool measures a single theoretical construct and is therefore unidimensional. To test the internal consistency of the tool’s reliability, a Cronbach’s alpha was computed as 0.84, indicating a high level of internal consistency for the assessment tool. All of the individual components would result in a lower Cronbach’s alpha if removed and none of the corrected item correlations were low (Figure 4). This suggests that none of the items should be removed from the assessment tool.

A G-theory study was performed to examine factors of: student, occasion and component of the assessment tool. The G-theory study revealed a co-efficient G (relative) of 0.90 and a co-efficient G (absolute) of 0.90, suggesting good global reliability. The study revealed that the principle sources of variance and possible error were students, which accounted for 27% of the overall variance in scores, and students nested in occasions, which accounted for a further 21.6% of the variance.

## Discussion

The literature identified how student nurses often feel ill-prepared for their leadership role as a newly qualified nurse (Ekstrom & Idvall, 2015). While attempts have been made to integrate leadership and management education into programmes (Francis-Shama, 2016; Ha & Pepin, 2018), concerns about prioritisation (Hendry & Walker, 2004) and the use of resources (Leal et al., 2017) persist. The role of placement learning has been acknowledged (ibid.) but students describe how differences in role models often negatively influence their views of leadership in the clinical setting (Francis-Shama, 2016). While regulators (Canadian Council for Registered Nurse Regulators, 2018; Nursing and Midwifery Board of Australia, 2006; Nursing and Midwifery Council, 2018; Singapore Nursing Board, 2018) have developed leadership and management competencies, they often do not prescribe how such experience should be gained. The development of the LMC assessment tool aimed to provide both a valid and reliable assessment of some of the leadership and management competencies associated with care delivery in a team, ward or department, and to standardise this experience across all students.

The content of the LMC tool was specifically selected, as it relates to the common attributes and behaviours that individuals would be expected to display day-to-day. While the literature review identified other concepts such as managing conflict and direction setting, these were not regarded as daily occurrences. The tool was designed to be easy to use and, prior to implementation, practice educators were trained in its use. The most significant change was the inclusion of a scale of competence. Prior to this tool, students were marked as having achieved or not achieved, or not addressed, for each of the NMC proficiencies. The scale of competence represented a significant change, as it moved staff from using a norm-referenced assessment, where they judged the student against the stage of the programme, towards a criterion referenced assessment (Turnbull, 1989), where the student is judged against the standard for registration. In addition to the training of practice educators, the reliability of assessment is improved by the addition of behavioural anchors to the scale. These descriptors of the level of supervision and support the student requires assist the practice educator in making decisions about the student’s level of competence.

The LMC tool allowed every student both to experience and be assessed in leading and managing a team, ward or department. This was achieved across a wide range of practice settings including critical care, community and acute wards. In some departments, such as critical care and the emergency room, a newly qualified nurse would never be in sole charge of the department, so these students were assessed leading a team within a larger department. The LMC tool was successfully implemented across a range of fields of practice including adult, children’s nursing and mental health. One issue noted with the LMC assessment tool was the timing of assessments, with an average of three days between the first and second assessment. This leaves little opportunity to act on feedback and to refine skills before the second assessment. It could addressed by being more prescriptive about when the first assessment can take place and allowing a two-week gap between the first and any subsequent assessment. Using LMC both formatively and summatively would also assist students to develop their skills and experience prior to any high-stakes summative assessment.

The LMC assessment showed good internal consistency and was identified as a reliable tool through both reliability analysis and using generalisability theory. The exploratory factor analysis revealed that all of the factors in the tool were loaded onto a single component, suggesting that the tool is unidimensional. This is interesting, given that it could be argued that communication, risk, delegation, prioritisation and the management of resources are not a single construct. Arguably, while the tool is designed to assess the single construct of clinical nursing leadership, the elements of risk, delegation, prioritisation and resource management might be seen as separate constructs. However, since ‘clinical leadership’ is already noted as being not well-defined (Cook, 2001), the need for some degree of consolidation is perhaps justified. One explanation might be that the practice educators are applying a ‘global rating’ of each student’s performance and are therefore considering each element globally as part of leading and managing a team. None of the elements of the tool is performed in isolation while leading; communication for example is integral to delegation and delegation is a key component of managing resources and so on. Global rating scales have been used in medicine as a replacement for checklists (Ma et al., 2012) and in other health professions alongside individual item ratings (Bremer et al., 2020). Ilgen et al. (2015) describe how global rating scales are more discriminating because they allow the input and analysis of information from various data points. A global rating is useful in this context because it mirrors how practitioners are required to practice by integrating various skills, knowledge and behaviour (Panzarella & Manyon, 2007). Further research is need to explore the role of global rating in the assessment of competence of nursing students.

## Strengths and Limitations

The LMC tool is easy to use and implement as a placement-based assessment of student nurse competence. The tool promotes the standardisation of opportunities to practice and be assessed in leading and managing a team, ward or department and, as such, enhances the student’s experience while at the same time enabling practice educators to assess competence in a more structured, valid and reliable way. This study was conducted with a single cohort and with a relatively small sample. As a preliminary evaluation, it has added to our understanding of some of the constructs that make up clinical nursing leadership. Further research to explore the potential use of global ratings by practice educators would be valuable.

In addition, the LMC tool has a number of limitations in that it only assesses 11 of the competencies that need to be assessed and signed off as achieved by the end of the pre-registration programme. However, integrated approaches to assessment, where a range of competencies are assessed together, is viewed as more beneficial than the assessment of single skills and attributes (Turnbull, 1989).

## Implications for Practice

LMC assessment provides nursing students with structured exposure to leadership and management opportunities during their practice placements. It also has the capacity to assist students to develop and enhance their skills using feedback and opportunities for deliberate practice (Bathish et al., 2018). The LMC tool should therefore support the student to make the transition to becoming a newly qualified nurse and reduce anxiety about skills around managing resources and prioritisation. As a competency assessment tool, LMC provides practice educators with a reliable method of assessing students prior to completion of their undergraduate programme. As a competency assessment tool, LMC provides practice educators with a reliable method of assessing students prior to completion of their under-graduate programme.

## Conclusion

LMC is a valid and reliable tool, which assesses the competence of student nurses to lead and manage care. As such, the tool addresses concerns expressed by both nurse educators and students about the degree to which students are prepared for their role in clinical nursing leadership, as they make the transition to becoming a newly qualified nurse. The tool is easy to use and, being paper based, students receive immediate feedback, including qualitative comments about performance and areas for improvement. In addition to its reliability, the tool can also provide opportunities for the development of structured educational experiences for students on placement.

## Appendix: Definitions of Each of the Constructs Developed by the Expert Panel

**Communication:** the transfer of clear and concise information both verbally and in writing between professionals and with patients and their families. During ‘hand off’ and when communicating urgent issues, the principles of SBAR (Situation, Background, Assessment and Recommendation) should be used.

**Managing resources:** includes both the safe and effective allocation of work to staff based on knowledge, skills and competence and the supervision of staff to ensure care is delivered in an effective and timely manner and that undue stress is avoided. In addition, non-human resources should be managed to avoid waste and to promote cost effective care delivery.

**Risk:** effectively identifies, assesses, and manages risk through controls. Advises others when patient and/or staff are at risk of harm. Maintaining appropriate records and reports including following reporting procedures and safeguarding policies.

**Prioritisation:** identification of priorities based on risk and communication of these to other staff. In addition, monitoring of care delivery to ensure priorities are met is an essential component of prioritisation.

**Delegation:** safe and effective delegation of work to others based on skill, knowledge and competence of the staff and care complexity. Delegation should allow for safe staff development and may include appropriate supervision.
